# Body Dysmorphic Symptoms Scale for patients seeking esthetic surgery: cross-cultural validation study: Escala de Sintomas da Dismorfia Corporal para pacientes que buscam a cirurgia plástica: estudo de validação cultural

**DOI:** 10.1590/1516-3180.2016.0068160416

**Published:** 2016-07-21

**Authors:** Tatiana Dalpasquale Ramos, Maria José Azevedo de Brito, Mônica Sarto Piccolo, Maria Fernanda Normanha da Silva Martins Rosella, Miguel Sabino, Lydia Masako Ferreira

**Affiliations:** I BSc. Master’s Student, Postgraduate Program on Translational Surgery, Universidade Federal de São Paulo (Unifesp), São Paulo, SP, Brazil.; II PhD. Affiliate Professor, College of Health Science, Universidade do Vale do Sapucaí (UNIVÁS), Minas Gerais; Postdoctoral Researcher, Division of Plastic Surgery, Department of Surgery, Universidade Federal de São Paulo (Unifesp), São Paulo, SP, Brazil.; III MD, PhD. Adjunct Professor, Postgraduate Program on Translational Surgery, Universidade Federal de São Paulo (Unifesp), São Paulo, SP, Brazil.; IV MD, PhD. Associate Professor, Division of Plastic Surgery, Department of Surgery, Universidade Federal de São Paulo (Unifesp), São Paulo, SP, Brazil.; V MD, PhD. Full Professor, Division of Plastic Surgery, Department of Surgery, Universidade Federal de São Paulo (Unifesp), São Paulo, SP, Brazil.

**Keywords:** Body dysmorphic disorders, Body image, Surgery, plastic, Psychiatry, Therapeutics, Transtornos dismórficos corporais, Imagem corporal, Cirurgia plástica, Psiquiatria, Terapêutica

## Abstract

**CONTEXT AND OBJECTIVE:**

Rhinoplasty is one of the most sought-after esthetic operations among individuals with body dysmorphic disorder. The aim of this study was to cross-culturally adapt and validate the Body Dysmorphic Symptoms Scale.

**DESIGN AND SETTING:**

Cross-cultural validation study conducted in a plastic surgery outpatient clinic of a public university hospital.

**METHODS:**

Between February 2014 and March 2015, 80 consecutive patients of both sexes seeking rhinoplasty were selected. Thirty of them participated in the phase of cultural adaptation of the instrument. Reproducibility was tested on 20 patients and construct validity was assessed on 50 patients, with correlation against the Yale-Brown Obsessive Compulsive Scale for Body Dysmorphic Disorder.

**RESULTS:**

The Brazilian version of the instrument showed Cronbach’s alpha of 0.805 and excellent inter-rater reproducibility (intraclass correlation coefficient, ICC = 0.873; P < 0.001) and intra-rater reproducibility (ICC = 0.939; P < 0.001). Significant differences in total scores were found between patients with and without symptoms (P < 0.001). A strong correlation (r = 0.841; P < 0.001) was observed between the Yale-Brown Obsessive Compulsive Scale for Body Dysmorphic Disorder and the Body Dysmorphic Symptoms Scale. The area under the receiver operating characteristic curve was 0.981, thus showing good accuracy for discriminating between presence and absence of symptoms of body dysmorphic disorder. Forty-six percent of the patients had body dysmorphic symptoms and 54% had moderate to severe appearance-related obsessive-compulsive symptoms.

**CONCLUSIONS:**

The Brazilian version of the Body Dysmorphic Symptoms Scale is a reproducible instrument that presents face, content and construct validity.

## RESUMO

**CONTEXTO E OBJETIVO:**

Rinoplastia é uma das operações mais procuradas por indivíduos com o transtorno dismórfico corporal. O objetivo deste estudo foi adaptar culturalmente e validar a Body Dysmorphic Symptoms Scale.

**DESENHO E LOCAL:**

Estudo de validação cultural desenvolvido no ambulatório de cirurgia plástica de um hospital universitário público.

**MÉTODOS:**

Oitenta pacientes consecutivos de ambos os gêneros que desejavam submeter-se à rinoplastia foram selecionados entre fevereiro de 2014 e março de 2015. Trinta pacientes participaram da fase de adaptação cultural do instrumento. A reprodutibilidade foi testada em 20 pacientes e a validade de construto em 50 pacientes, correlacionando-se a escala com a Yale-Brown Obsessive Compulsive Scale para transtorno dismórfico corporal.

**RESULTADOS:**

A versão brasileira do instrumento mostrou alfa de Cronbach de 0,805 e excelente reprodutibilidade interobservador (coeficiente de correlação intraclasse, CCI = 0,873; P < 0,001) e intraobservador (CCI = 0,939; P < 0,001). Houve diferença significante entre os escores totais de pacientes com e sem sintomas (P < 0,001). Observou-se forte correlação (r = 0,841; P < 0,001) entre a Yale-Brown Obsessive Compulsive Scale para transtorno dismórfico corporal e a Body Dysmorphic Symptoms Scale. A área sob a curva característica de operação do receptor (ROC) foi de 0,981, revelando boa acurácia para discriminar a presença de sintomas para transtorno dismórfico corporal; 46% dos pacientes apresentaram sintomas do transtorno dismórfico corporal e 54% dos pacientes apresentaram sintomas obsessivo-compulsivos moderados a graves relacionados com a aparência.

**CONCLUSÃO:**

A versão brasileira da Body Dysmorphic Symptoms Scale é um instrumento reprodutível que apresenta validade de face, conteúdo e construto.

## INTRODUCTION

More than 221,000 rhinoplasty procedures (or nose operations) were performed worldwide in 2013, mainly among Caucasians; about 163,600 of these procedures were performed on women.^1^ Rhinoplasty is often sought by young people between 13 and 34 years of age.^1^^,^^2^^,^^3^^,^^4^^,^^5^ Patients between 13 and 19 years account for 5% of all surgical cosmetic procedures performed.^1^^,^^2^ This shows the high level of social acceptance of esthetic surgery in general and of rhinoplasty in particular, as a means of physical enhancement in a culture in which physical attractiveness is highly valued, thus leading to greater concern regarding appearance based on an ideal standard body.^4^ However, the social importance of physical appearance also makes it difficult to diagnose body dysmorphic disorder.^4^

According to the Diagnostic and Statistical Manual of Mental Disorders, Fifth Edition (DSM-V), body dysmorphic disorder can be described as preoccupation with one or more perceived defects or flaws in physical appearance that are not observable or appear slight to other people, and compulsive or repetitive behavior (e.g. checking one’s appearance in a mirror, excessive grooming, skin picking and seeking reassurance) or mental acts (e.g. comparing one’s appearance with that of others) in response to concerns regarding appearance. It causes clinically significant distress or impairment in important areas of functioning, with symptoms that are poorly explained by normal concerns regarding physical appearance or by concerns regarding body fat or weight, among individuals meeting diagnostic criteria for eating disorders. Body dysmorphic symptoms may be associated with muscle dysmorphia. Patients with body dysmorphic disorder may show different degrees of insight regarding their body.^4^^,^^6^

Rhinoplasty is one of the most sought-after esthetic surgical procedures. Typical candidates include people with ethnically characteristic noses, teenagers and individuals with body dysmorphic disorder,^3^^,^^4^^,^^5^^,^^7^^,^^8^^,^^9^^,^^10^^,^^11^ which thus shows the social aspect of rhinoplasty. Rhinoplasty improves appearance through enhancing facial harmony. The inherent risks associated with the surgical process include respiratory problems, visible or palpable irregularities and dissatisfaction with the final outcome. Individuals with psychological or neurobiological vulnerability are more likely to show dissatisfaction with the surgical results, because their perception of the physical defect may be a symptom or contributory factor for development of a mental disorder.^3^^,^^4^ Rhinoplasty is also one of the cosmetic surgical procedures most frequently involved in lawsuits.^5^^,^^7^^,^^9^^,^^10^^,^^12^^,^^13^^,^^14^

Despite indications of improvement in psychosocial wellbeing following rhinoplasty, the prevalence of body dysmorphic disorder in patients seeking this surgical procedure ranges from 12% to 33%^10^^,^^13^^,^^15^^,^^16^^,^^17^^,^^18^ and 52%.^4^ Although the prevalence of psychiatric disorders among rhinoplasty patients seems inconsistent in the literature and requests for rhinoplasty should not be considered to be a symptom of a psychiatric disorder, screening for psychological conditions in selecting candidates for surgery is essential for a successful surgical cosmetic outcome.^4^^,^^19^^,^^20^^,^^21^^,^^22^

Excessive concern for appearance may conceal psychopathological states that are not always easily identified and which may lead to iatrogenic and medico-legal problems if neglected.^20^^,^^22^ The Body Dysmorphic Symptoms Scale is a specific instrument that measures psychopathological symptoms of body dysmorphic disorder.^23^ It is a short and easy-to-administer scale that captures specific information about body dysmorphic symptoms. Thus, cross-cultural validation of this patient-reported outcome measurement may help in relation to rapid screening for and identification of body dysmorphic disorder. Psychological disorders may not only affect the emotional and social life of patients, but also influence their satisfaction with the results from surgery.^24^^,^^25^

## OBJECTIVE

To translate into Brazilian Portuguese, culturally adapt and validate the Body Dysmorphic Symptoms Scale, by testing the psychometric properties, reproducibility and validity of the instrument, and to assess body dysmorphic disorder and levels of obsessive-compulsive symptoms among patients seeking esthetic surgery.

## METHODS

This cross-cultural validation study was approved by our institution’s Research Ethics Committee (approval no. 428.965/13) and was conducted in accordance with the Brazilian Ethical Review System for research involving human beings. It also conformed to the World Medical Association’s Declaration of Helsinki (June 1964) and subsequent amendments. Written informed consent was obtained from all patients or their parents or legal representatives after the procedures had been fully explained to them and prior to their inclusion in the study; anonymity was assured.

Patients of both sexes at any age, seeking rhinoplasty and showing physical appearance associated with clinically significant subjective distress, were recruited at the plastic surgery outpatient clinic of a public university hospital in Brazil between February 2014 and March 2015. A psychologist with expertise in body dysmorphic disorder, who was also one of the authors of this study, performed the clinical assessment on all patients, in accordance with the descriptions in the Diagnostic and Statistical Manual of Mental Disorders, Fifth Edition (DSM-V).^6^

Patients who were unable to understand the interview questions, those with severe physical deformities as a result of obesity, bariatric surgery, tumors or other conditions, those with psychotic disorders or previous history of body dysmorphic disorder, and those who had undergone psychiatric or psychological treatment were not included in the study.

The traditional protocol for determining an adequate sample size based on power analysis is not useful when the primary hypothesis focuses on psychometric measurement properties.^26^ A sample size of at least 50 and not more than 100 subjects is adequate for representing and evaluating the psychometric properties of social construct measurements.^26^ Thus, a total of 80 consecutive patients who met the study criteria were selected, of whom 30 participated in the cultural adaptation of the scale; 20 were included in the reliability analysis on the final version of the instrument; and these 20, together with 30 different patients, participated in the construct validity assessment against the Brazilian-Portuguese version of the Yale-Brown Obsessive Compulsive Scale for Body Dysmorphic Disorder. No patient declined to participate.

The cultural adaptation, reliability and validity phases of the study followed the methodology of Guillemin et al.^27^^,^^28^^,^^28^^,^^29^ and Gandek and Ware.^30^

The psychologist with expertise in body dysmorphic disorder also applied the cross-culturally validated Brazilian-Portuguese version of the Yale-Brown Obsessive Compulsive Scale for Body Dysmorphic Disorder to patients participating in the construct validity study.^31^

The Yale-Brown Obsessive Compulsive Scale for Body Dysmorphic Disorder is a 12-item semi-structured clinician-rated instrument that is designed to measure severity of body dysmorphic disorder symptoms among individuals showing excessive preoccupation and subjective distress with physical appearance.^31^ It is an outcome measurement for clinical studies and for treating body dysmorphic disorder.^32^ The 12 items are rated on a 0-4 scale, where 0 indicates no symptom and 4 indicates extreme body dysmorphic symptoms. The first 10 items assess excessive preoccupation, obsessions and compulsive behavior associated with dissatisfaction with physical appearance. The first three items are based on the body dysmorphic disorder diagnostic criteria and assess preoccupation, impairment of overall functioning, and subjective distress, which is related both to excessive preoccupation and to compulsive behavior. Items 11 and 12 assess insight and avoidance, respectively. The total score is calculated as the sum of ratings for the 12 items, thus yielding a maximum score of 48.^31^ The cutoff score of 19 for the Yale-Brown Obsessive Compulsive Scale for Body Dysmorphic Disorder has been correlated with sensitivity of 0.865 and specificity of 0.731.^31^

### The Body Dysmorphic Symptoms Scale

The Body Dysmorphic Symptoms Scale is a 10-item self-report measurement of psychopathological symptoms of body dysmorphic disorder among people with excessive concern and anxiety about their physical appearance who seek cosmetic surgery.^23^ The following are examples of the items: “Are you seriously concerned that one part of your body is defective?”, “Do you avoid looking at yourself in the mirror to be less worried?” and “Do you try to hide or camouflage your defect with your hands, hair, makeup, or clothing?” Each item is answered “yes” or “no”. The overall score is the sum of positive responses. High scores indicate the presence of psychopathological factors associated with dissatisfaction with body image and symptoms of body dysmorphic disorder.^23^

The present study was conducted after Dr. Perugi, the main author of the original version of the Body Dysmorphic Symptoms Scale,^23^ granted us permission to translate, culturally adapt and validate the instrument for Brazilian Portuguese.

### Translation

The Body Dysmorphic Symptoms Scale was translated from English into Brazilian Portuguese by two independent translators. Only one of the translators was informed about the study objectives, so as to achieve a conceptual rather than a literal translation of the scale. Both translations were evaluated by a multidisciplinary committee formed by two plastic surgeons, a psychiatrist and two psychologists with extensive experience of body image disorder and selection of candidates for cosmetic surgery. All items were checked by the multidisciplinary committee for possible mistakes made during the translation and were evaluated for content validity. A consensus Brazilian-Portuguese version of the instrument was then obtained by combining elements from both translations.^27^

Idiomatic, semantic, conceptual and cultural equivalences were considered during the translation phase. The consensus version in Brazilian Portuguese was then back-translated into English by two independent translators who were unaware of the original tool or purpose of the study. Both back-translated versions were evaluated and compared with the original one by the same multidisciplinary committee, in order to correct possible errors or discrepancies made during back-translation.^28^ This analysis resulted in development of the consensus version of the Body Dysmorphic Symptoms Scale in Brazilian Portuguese, which was appropriately adapted to the linguistic and cultural context of the target population, while maintaining all the essential characteristics of the original instrument in English.^29^

### Cross-cultural adaptation or pretesting

During the cultural adaptation phase, a psychologist with a doctoral degree and expertise in body dysmorphic disorder administered the consensus version of the Body Dysmorphic Symptoms Scale to the first 10 candidates for rhinoplasty and supervised a second psychologist during application of the instrument to the next 20 candidates. Interviews were conducted face to face. The cultural adaptation phase served to train the second psychologist for the inter-rater reliability phase.

The Body Dysmorphic Symptoms Scale was administered to 30 patients to test possible failures of the respondents to comprehend the items. After providing informed consent, the participants each had the opportunity to express their comprehension of the scale and suggest any changes that they considered necessary. All of the patients understood that the scale items were related to concerns and dissatisfaction with physical appearance.

In this phase, the face and content validity of the instrument were determined through a consensus reached by the multidisciplinary committee. Face validity evaluates whether the instrument measures what it was designed to measure and content validity relates to the degree to which each item is relevant in measuring the target content.^30^^,^^33^ The final version (Appendix 1) was obtained when the patients, translators and healthcare professionals reached a consensus.^29^^,^^34^

### Psychometric evaluation

After translation and cultural adaptation, the final version of the instrument was tested for reliability among 20 patients and for construct validity among the 20 patients together with 30 different patients, for a total of 50 patients.

### Reliability

Test-retest reliability (reproducibility) is the ability of an instrument to produce stable or similar results from repeated administration when no change to the patient characteristics has occurred. It evaluates the extent to which variation in scores between assessments reflects real differences rather than random fluctuation.^30^^,^^33^

The instrument was assessed by means of test-retest procedures in three interviews conducted by two independent interviewers (two experienced psychologists). Twenty patients were interviewed by psychologist #1 and the interview was repeated three hours later on the same day by psychologist #2. Two weeks later, the instrument was administered to the same patients by psychologist #1 only. Inter and intra-rater reliability analyses were performed. This phase of testing was used to verify the precision of the instrument for measuring the properties for which it was designed.^28^^,^^29^

### Validity

Construct validity is the process in which the correlation of a measurement with other variables is tested for theoretical consistency. In determining the construct validity, hypothesis testing indicates the direction and strength of the expected relationship.^30^^,^^33^ Our hypothesis was that preoccupation with physical appearance and excessive levels of body investment, together with clinically significant distress, among patients seeking cosmetic surgery, may be associated with symptoms of body dysmorphic disorder, which may be present at different levels of severity. Construct validity was assessed among 50 patients (20 patients who participated in the reliability analysis together with 30 different patients) using convergent and discriminant validity analyses. Convergent validity was tested by correlating the Body Dysmorphic Symptoms Scale with the Yale-Brown Obsessive Compulsive Scale for Body Dysmorphic Disorder scores. Discriminant validity was determined by comparing the mean Body Dysmorphic Symptoms Scale scores of patients with and without body dysmorphic disorder symptoms.

A cutoff point for symptom severity and the corresponding sensitivity and specificity values were estimated through the receiver operating characteristic curve, which was constructed based on the clinical evaluation of body dysmorphic disorder, in accordance with the descriptions in the Diagnostic and Statistical Manual of Mental Disorders, Fifth Edition.

### Statistical analysis

Cronbach’s alpha was used to evaluate the internal consistency of the reliability of the instrument.

Test-retest reliability and convergent validity were estimated using Pearson’s correlation coefficient (*r*) and the intraclass correlation coefficient (ICC).

Discriminant validity was determined using Student’s t-test for independent samples.

A cutoff point for symptom severity and the corresponding sensitivity and specificity values were estimated through the receiver operating characteristic curve. The kappa coefficient was also calculated.

The Statistical Package for the Social Sciences version 20.0 (SPSS Inc., Chicago, IL, USA) and Stata 12 software (StatCorp, College Station, Texas, USA) were used for data analysis. All statistical tests were performed at a significance level of 5% (P < 0.05). Data were expressed as mean ± standard deviation (SD).

## RESULTS

**Figure 1.**
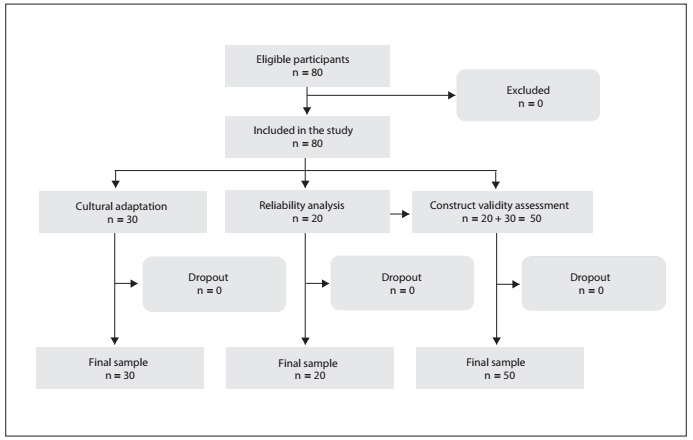
Flow diagram showing the initial recruitment and final sample of patients.

The Brazilian-Portuguese version of the Body Dysmorphic Symptoms Scale (Appendix 1) was administered to 80 patients. The flow diagram showing the initial recruitment and the final sample of patients is shown in Figure 1. The patients did not have any doubts about the items, which were considered easy to understand and clearly formulated. The mean time taken to respond to the questionnaire was five minutes.

Thirty-seven patients (37/80; 46%) met the diagnostic criteria for body dysmorphic disorder, according to the Body Dysmorphic Symptoms Scale, and 27 patients (27/50; 54%) showed moderate to severe appearance-related obsessive-compulsive symptoms.

The mean Body Dysmorphic Symptoms Scale score was 7.5 ± 1.0 (range, 6-9; t = 12.3; P < 0.001).

Overall, most patients were women (80%), Caucasians (75%) and single (58.8%). The mean age was 33.4 ± 11.8 years (range, 14-65); 55.1% reported spending three or more hours a day concerned about their physical appearance and 79% of patients reported that they began to experience body dissatisfaction during childhood and adolescence. Thus, the time that elapsed from the onset of body dissatisfaction to the patient’s decision to seek cosmetic treatment was about 15 years. Also, 52.5% had completed high school education and 21% were semi-skilled workers.

The instrument showed good internal consistency (Cronbach’s alpha = 0.805). All items contributed favorably towards the internal consistency of the scale (Table 1).

**Table 1.**
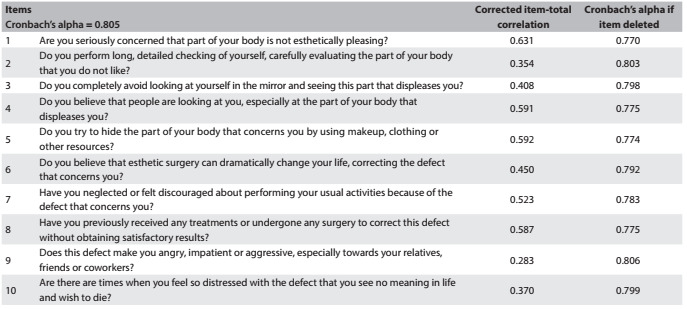
Internal consistency analysis for the Body Dysmorphic Symptoms Scale (n = 80)

The corrected item-total correlation was greater than 0.4, except for items 2, 9 and 10, thus indicating that the consistency between item scores and the overall score of the instrument was acceptable (Table 1).

According to the Body Dysmorphic Symptoms Scale, 56 patients (70%) reported that they compulsively checked their appearance in a mirror; 54 (67.5%) often tried to camouflage the perceived defect with their hands, hair or excessive makeup; 65 (81.3%) had previously sought esthetic surgical procedures; 30 (37.5%) were dissatisfied with the results from the previous esthetic surgery; 56 (70%) showed self-referential perceptions due to exaggeration of the perceived defect; and 54 (67.5%) had poor insight regarding their perceived defects, believing that they had real physical deformities for which esthetic surgery was needed. Psychosocial impairment was identified in 25 patients (31.3%), who avoided affective and social relationships; while 33 patients (41.3%) avoided looking in the mirror, thus showing aversion to their own image. Six patients (7.5%) showed aggressive and violent behavior towards their relatives and friends, and 12 (15%) were so distressed that they were at the point of having suicidal thoughts.

The Body Dysmorphic Symptoms Scale demonstrated excellent inter-rater reliability (r = 0.909; ICC = 0.873; P < 0.001) and intra-rater reliability (r = 0.956; ICC = 0.939; P < 0.001), as seen in Table 2.

**Table 2.**
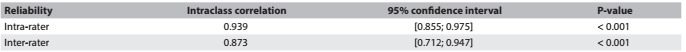
Inter and intra-rater reliability for the Body Dysmorphic Symptoms Scale

**Figure 2.**
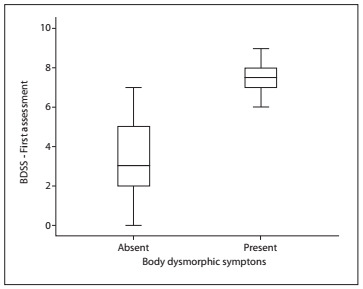
Distribution of patients with and without body dysmorphic symptoms, according to the Body Dysmorphic Symptoms Scale (BDSS).

There were significant differences in Body Dysmorphic Symptoms Scale scores between patients with and without body dysmorphic symptoms (P < 0.001). Patients without body dysmorphic symptoms had significantly lower Body Dysmorphic Symptoms Scale scores than those with body dysmorphic symptoms (Figure 2).

**Figure 3.**
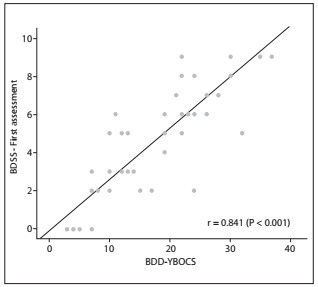
Correlation between the Body Dysmorphic Symptoms Scale (BDSS) and the Yale-Brown Obsessive Compulsive Scale for Body Dysmorphic Disorder (BDD-YBOCS).

A strong positive correlation (r = 0.841; P < 0.001) was found between the Body Dysmorphic Symptoms Scale and the Yale-Brown Obsessive Compulsive Scale for Body Dysmorphic Disorder (Figure 3).

**Figure 4.**
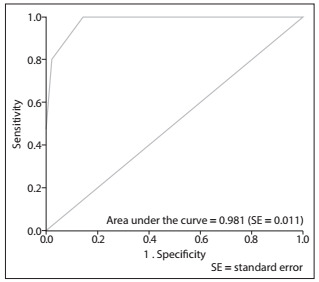
Receiver operating characteristic curve for the Brazilian version of the Body Dysmorphic Symptoms Scale.

A cutoff score of 6 was determined for the Body Dysmorphic Symptoms Scale using the receiver operating characteristic curve (Figure 4); this was associated with sensitivity of 1.0 and specificity of 0.86. Scores of 6 and above indicate the presence of psychopathological characteristics that were associated with dissatisfaction with body image and symptoms of body ­dysmorphic disorder. The area under the receiver operating characteristic curve (ROC) was 0.981, thus suggesting that the Body Dysmorphic Symptoms Scale presented very good accuracy for discriminating between presence and absence of body dysmorphic symptoms.

The kappa coefficient between the Yale-Brown Obsessive Compulsive Scale for Body Dysmorphic Disorder (for a cutoff point of 19) and the Body Dysmorphic Symptoms Scale (for a cutoff point of 6) was 0.721, thus showing that there was strong agreement between the cutoff points for severe body dysmorphic symptoms.

The final Brazilian version of the instrument was named Body Dysmorphic Symptoms Scale-Unifesp-EPM or BDSS-Unifesp-EPM (Escala de Sintomas da Dismorfia Corporal - Unifesp-EPM, in Brazilian Portuguese).

## DISCUSSION

The Body Dysmorphic Symptoms Scale was translated into Brazilian Portuguese, culturally adapted and tested for reliability and construct validity. The general guidelines for cross-cultural adaptation of instruments were followed in order to ensure the quality of the cross-culturally adapted Brazilian version of the Body Dysmorphic Symptoms Scale (Appendix 1). Healthcare professionals who were experienced in managing patients with body dysmorphic disorder and rhinoplasty patients participated in the evaluation on this instrument.^27^

The Brazilian-Portuguese version of the Body Dysmorphic Symptoms Scale was validated in a population sample of 80 cosmetic surgery patients and showed excellent internal consistency, test-retest reliability and intra-rater reliability. However, it was not possible to compare these results with those of the original scale or with the scientific literature because the psychometric properties of the scale were not assessed by the authors of the instrument,^23^ or by Mühlbauer et al.,^35^ who proposed a modification of item 6 regarding unrealistic expectations and called the instrument the Modiﬁed Pisa Body Dysmorphic Symptoms Scale.

The psychometric properties of the Body Dysmorphic Symptoms Scale were evaluated for the first time in the present study. A cutoff score of 6, which was determined using the receiver operating characteristic curve, was able to discriminate between patients with body dissatisfaction and those with body dysmorphic disorder. The cutoff score of 6 was associated with sensitivity of 1.0 and specificity of 0.86, thus indicating that the Brazilian version of the Body Dysmorphic Symptoms Scale is a specific instrument for identifying body dysmorphic symptoms. This tool may be used preoperatively, in screening the candidates for esthetic surgery procedures.

In order to assess construct validity, it is recommended in the literature that the instrument should be compared against a similar tool, so as to evaluate the relationships of comparable constructs with similar operational concepts.^30^ Thus, the Body Dysmorphic Symptoms Scale was compared against the cross-culturally validated Brazilian-Portuguese version of the Yale-Brown Obsessive Compulsive Scale for Body Dysmorphic Disorder, which measures the degree of dissatisfaction with a given physical feature and the severity of body dysmorphic symptoms.^31^ The strong correlation between the two instruments indicates that the Body Dysmorphic Symptoms Scale was able to measure the severity of body dysmorphic symptoms, and that both instruments are able to detect patterns of neurocognitive deficits (obsessive thoughts and compulsive behavior) that are present in body dysmorphic symptoms. However, the Yale-Brown Obsessive Compulsive Scale for Body Dysmorphic Disorder is a semi-structured, longer and more complex tool that is designed to be applied by professionals who do not have much background within mental health, with regard to selecting patients who are seeking esthetic and surgical procedures, whereas the Body Dysmorphic Symptoms Scale is a short and easy-to-administer scale that also captures specific information about body dysmorphic symptoms.

The assessment of discriminant validity showed that there was a significant difference in mean Body Dysmorphic Symptoms Scale scores between patients with and without body dysmorphic symptoms. A larger number of patients reported high scores for items 1, 2, 4, 5 and 6, thus showing dissatisfaction with their body image with regard to compulsive behavior (e.g. checking their appearance in a mirror and excessive grooming) and mental acts (e.g. comparing their appearance with that of others) in combination with subjective distress, which are the factors that most interfere with the overall functioning of patients with body dysmorphic disorder. The levels of subjective distress and psychosocial impairment that are associated with physical appearance may be the most important parameters to be assessed among cosmetic surgery patients.^22^ About 81% of the patients believed that cosmetic surgery would solve all their problems relating to the distress caused by their physical appearance (item 6), and 67% of the patients were convinced that a perceived defect was really present and had fixed ideas about their perception (item 1). This belief appeared to be related to exaggeration of the defect rather than to a delusional perception, but in 70% of the patients it enhanced self-referential ideas (item 4).^4^^,^^19^

Items 2, 9 and 10 of the Body Dysmorphic Symptoms Scale presented corrected item-total correlation values of less than 0.4, which suggested that these items had a weak correlation with the other items of the scale. This may have related to the presence of body dysmorphic symptoms (as described in the Diagnostic and Statistical Manual of Mental Disorders, Fifth Edition, in the diagnostic criteria for body dysmorphic disorder A and B) in this population (item 2), and may have indicated that the patients in this study did not have any auto or hetero-aggressive behavior (items 9 and 10). In fact, 70% of the patients responded positively to item 2 and only 7.5% and 15% responded positively to items 9 and 10, respectively, which were the items with the lowest scores in the instrument.

The prevalence of body dysmorphic symptoms was 46% in the study sample (according to the Body Dysmorphic Symptoms Scale), and 54% of the patients had moderate to severe appearance-related obsessive-compulsive symptoms, according to the Yale-Brown Obsessive Compulsive Scale for Body Dysmorphic Disorder. Most of the patients began to experience body dissatisfaction during childhood and adolescence, and were spending three or more hours a day on appearance-related concerns and behavior, and showed higher levels of subjective distress. The fact that 58.8% of the patients were single, 52.5% had only completed secondary education and 21% were semi-skilled workers may suggest that the disorder caused psychosocial impairment over time among these patients. Picavet et al.^13^ identified moderate to severe appearance-related obsessive-compulsive symptoms in 33% of their patients seeking rhinoplasty, also using the Yale-Brown Obsessive Compulsive Scale Modified for Body Dysmorphic Disorder. The high prevalence of body dysmorphic symptoms found in the present study is similar to those found in previous studies.^3^^,^^4^

The participants’ mean age was 33 years at the time of the interview, which was not associated with the onset of body dysmorphic symptoms and thus was consistent with the literature.^1^^,^^2^^,^^3^^,^^4^^,^^5^^,^^13^^,^^23^ The time that elapsed from the onset of body dissatisfaction to the patient’s decision to seek cosmetic treatment (about 15 years) was very similar to that of patients seeking mental health treatment, thus showing the different behaviors and profiles of this population.^36^ In other words, patients with body dysmorphic disorder may take different paths; those who seek cosmetic surgery will not necessarily seek psychiatric treatment later.^36^ Most of the patients were women and Caucasians, which is in agreement with previous studies.^1^^,^^4^

The limitations of this study include its small sample size and the fact that most of the patients were women. In addition, the study was conducted on a clinical population that usually has greater disease severity, given that higher rates of disease severity have been observed in clinical samples than in the general population.^37^^,^^38^^,^^39^ This may have affected the cutoff score on the Body Dysmorphic Symptom Scale, which may be different in other situations. Further studies with a larger number of patients and involving multiple centers are necessary in order to evaluate and compare the prevalence of body dysmorphic symptoms among patients seeking plastic surgery, so as to enable development of care and treatment strategies for this population.

## CONCLUSION

The cross-culturally validated Brazilian-Portuguese version of the Body Dysmorphic Symptoms Scale is a reliable instrument that shows face, content and construct validity. It is a useful tool that can contribute towards screening candidates with body dysmorphic disorder for cosmetic surgery. The prevalence of moderate to severe body dysmorphic and appearance-related obsessive-compulsive symptoms is high among patients seeking esthetic rhinoplasty.
